# Probing the content of semantic representations in body-selective regions

**DOI:** 10.1162/IMAG.a.1309

**Published:** 2026-07-27

**Authors:** Ryuto Yashiro, Masataka Sawayama, Ayumu Yamashita, Kaoru Amano

**Affiliations:** Graduate School of Information Science and Technology, The University of Tokyo, Tokyo, Japan; Department of Education and Psychology, Freie Universität Berlin, Berlin, Germany; Institute of Cognitive Science, Universität Osnabrück, Osnabrück, Germany; Graduate School of Information Science and Technology, Hokkaido University, Sapporo, Hokkaido, Japan; Prometech CG Research, Tokyo, Japan

**Keywords:** body-selective regions, encoding models, semantic representation, co-occurrence, implied motion

## Abstract

Recent advances in neural networks trained on natural language have revealed that category-selective regions encode complex semantics and contextual information of natural scenes in addition to object categories. However, the limited interpretability of embeddings derived from these models complicates the characterization of the aspects of natural scenes that contribute to such semantic representations. Here we addressed this question by developing an analysis of the relationship between object co-occurrence in large-scale natural scene captions and corresponding fMRI responses predicted by caption-based encoding models, inspired by the fact that the joint presence of multiple objects generally shapes the overall content of a scene. We performed this analysis on the extrastriate body area (EBA), which responds strongly to human body parts. We found that human bodies co-occurring with sports-related objects drive the strongest predicted responses in the EBA among all image categories, whereas those co-occurring with vehicles or accessories elicit strong but weaker predicted responses. The findings from the co-occurrence analysis helped identify three key body-related features that contribute to the semantic representation in the EBA and fusiform body area: human body motion speed implied in static images of natural scenes is a primary contributor, and the number of people and body size are secondary contributors. Our framework, integrating object co-occurrence with caption-based encoding models, offers an interpretable approach for understanding high-level visual representations underlying natural scene perception.

## 1 Introduction

Humans can recognize diverse natural scenes containing multiple objects. Previous studies have identified a set of specialized brain regions that appeared to selectively respond to biologically important constituents of those scenes, such as faces and bodies, in the late stages of hierarchical visual processing (Downing et al., 2001; Kanwisher et al., 1997). Although these category-selective regions were identified by measuring neural responses to segmented faces and body parts in well-controlled neuroimaging experiments, subsequent studies have proposed a more nuanced view of category selectivity, showing that these regions are not strictly selective for a single category, but also contain information about other categories and stimulus dimensions (Caldara & Seghier, 2009; Grill-Spector et al., 2006; Hanson et al., 2004; Haxby et al., 2001; O’Toole et al., 2005; Sinke et al., 2012; Slotnick & White, 2013).

The traditional view of category-selective regions has been further refined by naturalistic approaches (Kayser et al., 2004; Nastase et al., 2020), in which brain responses are measured using large-scale, more naturalistic visual stimuli, such as images and movies containing multiple people and objects (Allen et al., 2022; Hebart et al., 2023; Lahner et al., 2024). At the core of this naturalistic approach are encoding models that accurately predict brain responses to visual stimuli from a set of features derived from those stimuli (Kriegeskorte & Douglas, 2019; Naselaris et al., 2011). With the advent of neural networks trained with millions of images (Dosovitskiy et al., 2020; Krizhevsky et al., 2017), encoding models have achieved remarkable accuracy in predicting visually evoked responses, deepening our understanding of both how and to what extent stimulus-derived features are represented in distinct brain regions along the visual processing hierarchy (Bashivan et al., 2019; Gu et al., 2022; St-Yves & Naselaris, 2018). Recent studies in this direction have used internal representations of neural networks trained on large-scale images and accompanying text descriptions (captions) to construct encoding models of brain responses to natural scene images (Luo et al., 2024; Wang et al., 2023). These models with natural language supervision showed a stronger predictive capacity, particularly for high-level visual regions, than that of those with the same architecture but trained only on visual images (Wang et al., 2023). More recently, internal representations of large language models (LLMs) derived from natural scene captions, referred to as caption embeddings, have been used to construct encoding models that account for brain responses to natural scenes. These embeddings, which capture physical properties (e.g., color, shape, or spatial layout) as well as semantic properties (e.g., object category, animacy, or naturalness) of natural scenes, exhibited a high capacity to predict responses to natural scenes in the ventral temporal cortex (Doerig et al., 2025; Horikawa, 2025). Importantly, encoding models based on embeddings of full captions outperformed those based on embeddings derived from only the nouns of the captions (Doerig et al., 2025) or internal representations of deep neural networks pretrained on large-scale visual data (Horikawa, 2025). Therefore, high-level visual regions contain rich information regarding the meaning and context of natural scenes—information that is effectively captured by language—suggesting the presence of semantic representations in the category-selective regions that extend beyond a single object category.

Despite this new perspective of the neural representation in the category-selective regions, interpreting what aspects of natural scenes constitute the semantic representation remains challenging, because caption embeddings typically used in the high-performing encoding models reflect an entangled mixture of multiple physical and semantic aspects of natural scenes. To address this, we sought to gain deeper insight into the semantic representation in the category-selective regions by focusing on the fact that the co-occurrence of multiple objects in a scene generally shapes the semantic content of that scene. For example, a person sitting at a table with a fork and a dish may be interpreted as preparing to eat, whereas replacing those items with a laptop leads us to interpret the scene as someone working. Thus, identifying co-occurring object pairs that drive different levels of responses in category-selective regions could help us understand the content of their semantic representations.

**Fig. 1.**
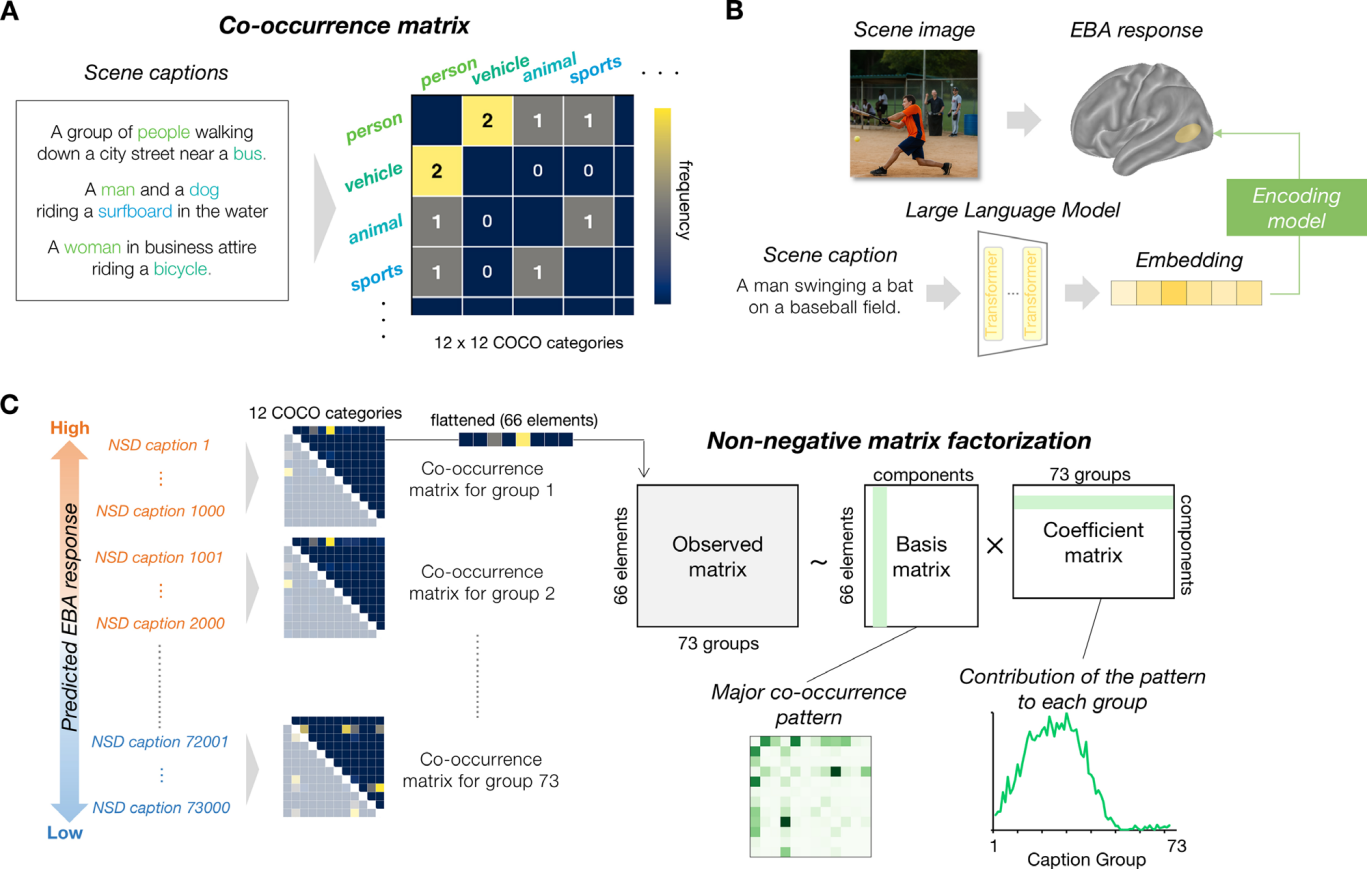
Illustration of our co-occurrence analysis comprising three key elements. (A) Co-occurrence matrix. In our framework, a co-occurrence matrix was defined by counting the co-occurrence frequency of all pairs of 12 MS-COCO superordinate categories in a set of captions that describe natural scene images. By exploring the relationship between the co-occurrence matrix derived from scene captions and EBA responses to those scenes, we sought to gain insights into what specific aspect of semantic content of natural scenes is associated with the representation in EBA. (B) Caption-based encoding model. We constructed ridge regression models using 9,000–10,000 pairs of image-derived captions and image-evoked responses for each of 8 NSD subjects. Captions were passed through a large language model (MPNet) into 768-dimensional vectors, and these embeddings were used as predictors in our encoding models. For copyright reasons, the example scene image is a generated image that preserves the semantic content and composition of the original COCO image. (C) Non-negative matrix factorization. Based on the predicted responses averaged across the 8 subjects, all captions were sorted and divided into 73 groups. For each group, a co-occurrence matrix was constructed. The 66 upper-triangular elements from each matrix were flattened and concatenated column-wise to form a single 66 × 73 matrix. We then performed non-negative matrix factorization to decompose the observed matrix into two matrices: basis and coefficient matrices. Each column of the basis matrix represents a co-occurrence pattern corresponding to one component when reshaped back into its original 2-dimensional form, while each row of the coefficient matrix (a coefficient vector) reflects how strongly each co-occurrence pattern is represented across the 73 groups. As the groups were sorted in descending order of predicted EBA response, each coefficient vector captures the relationship between the corresponding co-occurrence pattern and the magnitude of predicted EBA responses. The optimal number of components was determined by minimizing the Bayesian Information Criterion (BIC).

In this study, we focused on the semantic representation of the extrastriate body area (EBA), a major category-selective region that has been extensively characterized as a body-selective region in the human visual cortex (Downing et al., 2001; Downing & Peelen, 2011; Peelen & Downing, 2007; Urgesi et al., 2004; Vangeneugden et al., 2014). EBA provides an ideal test case for our co-occurrence-based approach because its responses depend on the presence of bodies as well as on contextual and action-related information embedded in natural scenes (Kable & Chatterjee, 2006; Takahashi et al., 2008; Wiggett & Downing, 2011), which can be naturally captured by the co-occurrence structure of objects. We developed an analysis that links object co-occurrence statistics in scene captions to EBA responses (Fig. 1). Specifically, using large-scale fMRI responses to natural images in the Natural Scenes Dataset (NSD) (Allen et al., 2022) along with Microsoft Common Objects in Context (MS-COCO) captions that describe those images (Chen et al., 2015; Lin et al., 2014), we examined what object categories co-occurring with human bodies in natural scenes are associated with EBA responses predicted by caption-based encoding models.

To preview our main findings, the encoding model in our co-occurrence analysis accurately predicted the strongest EBA responses among all image categories for images containing both people and sports-related objects, and predicted strong but slightly weaker EBA responses for images of people co-occurring with accessories or vehicles than for those co-occurring with sports-related objects. Combining these results with correlation analyses between EBA responses and several candidate image features, we found that the speed of dynamic body motion implied in static images constitutes a predominant component of semantic representations in the EBA, with the number of people and body size serving as secondary contributors. We argue that our framework, which combines co-occurrence analysis and caption-based encoding models, provides a promising approach for interpreting high-level visual representations.

## 2 Materials and Methods

### 2.1 Large-scale fMRI data

We utilized the NSD (Allen et al., 2022), which includes high-quality fMRI responses to a wide array of color natural scene images. These images are extracted from the MS-COCO database (Lin et al., 2014). Eight NSD subjects viewed 9,000–10,000 images while whole-brain fMRI data were acquired on a 7T scanner using gradient-echo EPI at 1.8-mm isotropic resolution with a repetition time (TR) of 1,600 ms and an echo time (TE) of 22 ms (see Allen et al., 2022 for acquisition details). Each image was repeatedly presented up to 3 times throughout 40 scan sessions. A set of 1,000 images was viewed by all subjects, and each subject additionally viewed 8,000–9,000 unique images that were not viewed by any other subject. Each image was 8.4° × 8.4° and was presented for 3 seconds. The subjects were asked to fixate on a centrally presented small dot and indicate by pressing a button whether they had seen each image before.

NSD provides whole-brain fMRI responses as beta values obtained from general linear modeling (GLMsingle) (Prince et al., 2022). We used beta values called fithrf_GLMdenoise_RR defined on the cortical native surface space of each subject, which were z-scored within each scan session. These beta values were generated using optimized denoising and regularization methods with vertex-specific hemodynamic response functions (HRFs), yielding more reliable response estimates compared with methods assuming a canonical HRF for all vertices (Allen et al., 2022; Prince et al., 2022). To restrict our analysis to a set of vertices with reliable signals, the noise ceiling signal-to-noise ratio (NCSNR) metric was used to subsample the vertices with NCSNR equal to or greater than 0.2, in accordance with a previous study (Conwell et al., 2024). After this subsampling process, we extracted response patterns for body-selective regions via the region of interest (ROI) definition and statistical contrast (t value) maps derived from functional localizer experiments in the NSD. Specifically, we defined the EBA and fusiform body area (FBA) as sets of vertices with t values equal to or greater than 1 to investigate whether these anatomically distinct body-selective regions represent different aspects of natural scenes. Given the partial overlap between motion-selective regions and the EBA (Ferri et al., 2013; Peelen et al., 2006; Weiner & Grill-Spector, 2011), we also defined the middle temporal (MT) and middle superior temporal (MST) areas for each subject, enabling us to extract EBA vertices located outside these motion-selective regions. This definition was based on the HCP atlas (HCP_MMP1) (Glasser et al., 2016), which was projected onto each subject’s native surface space by the preprocessing pipeline used in the NSD. We created masks for the MT and MST using the corresponding HCP atlas area names (MT and MST, respectively). Because the NSD did not include a localizer experiment for motion-selective regions, we used an atlas-based definition as an alternative. Because atlas-defined regions may not perfectly align with regions individually localized using functional localizer experiments, caution is warranted when interpreting the results for MT and MST. Unless otherwise specified, our analyses were based on the mean response computed across repetitions of each image presentation.

### 2.2 Co-occurrence analysis

To understand which aspects of natural scenes are critical for determining EBA responses, we constructed an encoding model using text descriptions of the images and embeddings of those descriptions obtained from an LLM (Doerig et al., 2025; Horikawa, 2025). We then investigated how the responses predicted by the model were associated with co-occurring word pairs in the captions. By using encoding models, we were able to make full use of the 73,000 caption–response pairs in the NSD, which far exceeds the 1,000 images shared across 8 subjects, enabling us to reliably extract general object co-occurrence patterns associated with EBA responses.

#### 2.2.1 Encoding models based on text descriptions of natural scene images

We trained an encoding model that predicts the EBA response to a natural scene image given the corresponding caption provided by a human annotator (Fig. 1B). Each NSD image has 5 captions (Chen et al., 2015), and each caption was fed into an LLM (all-mpnet-base-v2) (Song et al., 2020) to extract 768-dimensional embeddings via the Sentence-Transformers library. We then averaged these embeddings across five captions to obtain one embedding for each image, in accordance with a previous study (Doerig et al., 2025). The embeddings served as predictors, allowing us to construct an encoding model using fractional ridge regression (Rokem & Kay, 2020). This method differs from conventional ridge regression by parameterizing regularization with an interpretable fraction, rather than the usual abstract hyperparameter lambda. This fraction specifies the regularization strength relative to the unregularized solution, allowing efficient exploration of the full regularization range. We estimated the best linear weights and hyperparameter according to the following procedure: first, we held out as a test set the caption embeddings of 1,000 images that were viewed by all 8 subjects in the NSD experiment. The remaining embeddings, corresponding to 8,000–9,000 images uniquely observed by each NSD subject, were subsequently used as a training set. Within this training set, we fitted subject-specific ridge regression models using fivefold cross-validation and selected the regularization fraction that achieved the best mean validation performance across folds. We then refitted the model on the entire training set using the selected fraction. Fraction values ranged from 0.1 to 1 in steps of 0.1, where 1 indicates no regularization and 0 indicates complete regularization, with all weights shrunk to zero. The resulting encoding model was applied to the held-out embeddings to evaluate prediction accuracy by computing the Pearson correlation coefficient between the predicted and actual EBA responses averaged across repetitions for each image.

#### 2.2.2 Defining a co-occurrence matrix from captions

A co-occurrence matrix typically consists of nonnegative integer values corresponding to the frequency of a word pair co-occurring in large-scale sentences. To define a co-occurrence matrix for the captions of the NSD images, we used multiple object categories at the superordinate level in the MS COCO dataset and their co-occurrence frequency in a group of captions (Fig. 1A). Specifically, we constructed a 12 × 12 matrix based on the co-occurrence frequency of 12 superordinate categories (accessory, animal, appliance, electronic, food, furniture, indoor, kitchen, outdoor, person, sports, and vehicle) derived from 91 basic COCO categories (Lin et al., 2014), with 2 important modifications from the original category classification. First, we included several person-related words (man, woman, boy, girl, baby, child) as members of the “person” category. The reason for this modification is that these specific words are much more frequently used by human annotators to describe the presence of a person in images than the word “person”. Second, any words that constitute the four phrases included in the “sports” category (sports ball, baseball bat, baseball glove, and tennis racket) are also considered members of the “sports” category (e.g., “baseball” and “bat” from “baseball bat”) because these constituent words are more frequently used in captions than are the phrases themselves. These modifications resulted in 12 superordinate categories derived from 100 basic categories (see Supplementary Table S1 for details).

Using these modified COCO object categories, we constructed multiple co-occurrence matrices from the captions associated with the NSD images. The construction process involves several steps. In the first step, we extracted the first of the 5 captions for each NSD image and fed the corresponding LLM (all-mpnet-base-v2) embeddings into the trained encoding models for each of the 8 subjects, yielding predicted EBA responses to the 73,000 images for each subject (Fig. 1C, left). In the second step, we sorted the 73,000 captions in descending order of the predicted EBA responses averaged across subjects and partitioned them into 73 groups of 1,000 captions each, with the first group corresponding to the highest predicted EBA responses and the last to the lowest. The third step involves lemmatizing all nouns in the captions for each group using the WordNet lemmatizer in the nltk library, which converts inflected noun forms into their base form. This allowed us to accurately count all nouns of the 100 basic categories in the next step, regardless of their form in the captions. Finally, we counted the co-occurrence frequency of all 66 pairs of superordinate categories for the captions in each group, yielding 73 symmetric co-occurrence matrices that consisted of nonnegative integer values (Fig. 1C, middle).

To extract co-occurrence components closely linked to EBA responses in a data-driven manner, we performed Bayesian nonnegative matrix factorization on the data derived from the 73 co-occurrence matrices. Specifically, as the co-occurrence matrices are symmetric, we extracted their upper triangular portion, flattened it into a vector, and horizontally stacked the vectors to obtain a matrix of size 66 × 73 (Fig. 1C, right). This matrix was decomposed into two different matrices: basis and coefficient matrices. Each column in the basis matrix represents a major co-occurrence pattern that is most strongly represented in the original matrices, and each row in the coefficient matrix reflects the extent to which each co-occurrence pattern contributes to reconstructing the original matrix. By examining the 73 values in each row, we can assess how strongly each major co-occurrence pattern is associated with the 73 groups sorted based on EBA responses, thereby capturing its relationship with the magnitude of EBA responses. We performed this factorization while varying the number of components from 1 to 10 in increments of 1 and determined the optimal number of components as the one that minimizes the Bayesian information criterion (BIC). The BIC was defined as follows:

BIC=−2L+K(m+n)log mn, 

where m and n denote the number of rows and columns of the original matrix, respectively, and K indicates the number of components. We assumed a Poisson distribution for the co-occurrence count of each category pair because the co-occurrence matrices contained non-negative integer values with many zero entries. Under this assumption, the log-likelihood L was defined as follows:

$$L=\sum_{i=1}^{m}\sum_{j=1}^{n}\left(v_{ij}\log\sum_{k=1}^{K}w_{ik}h_{kj}-\sum_{k=1}^{K}w_{ik}h_{kj}-\log v_{ij}!\right),$$

where v, w, and h are individual elements of the observed, basis, and coefficient matrices, respectively.

### 2.3 Correlation analysis using body-related image features

The co-occurrence analysis revealed specific category pairs associated with EBA response magnitude. We then visually inspected representative images containing these category pairs to identify aspects of the scenes that could plausibly account for EBA responses, which led us to define six candidate features for subsequent quantitative analyses. To test this possibility, we computed the correlation between the features and brain responses across vertices in the EBA and FBA for each NSD subject, by using Spearman’s rank correlation for discrete features (implied motion and number of people), and Pearson correlation for continuous features (body size, face size, distance from the center, and root mean square (RMS) contrast). Definitions of all features are provided below, except for implied motion, which we collected in a behavioral experiment (see the next section for details). We expected positive correlations with EBA responses for implied motion, number of people, and body size, and negative correlations for the remaining features (see Results section for details). To evaluate the statistical significance of the correlation for each vertex, we performed a one-sided permutation test with 1,000 randomizations. For each randomization, we permuted the stimulus labels and computed a correlation, yielding a null distribution of correlation coefficients. P-values were defined as the proportion of correlations exceeding the observed correlation coefficient and were corrected for multiple comparisons using the Benjamini–Hochberg false discovery rate (FDR) procedure (*p* < 0.05).

#### 2.3.1 Body size

To compute the body size for each image, we used a text-based segmentation model called “Grounded Segment Anything” (T. Ren et al., 2024). This model leverages the strengths of two machine learning models: grounding DINO (Liu et al., 2023), which identifies the presence of an object specified by text input, and Segment Anything (Kirillov et al., 2023), which performs segmentation of objects and people within an image. For each of the 100 images used in the rating experiment, we segmented people by providing the text input “person” to the model and computed the human body size, which was defined as the number of pixels corresponding to the human body in each image. If multiple people were present in an image, we computed the total body size by summing the body sizes of all individuals.

#### 2.3.2 Face size

Faces were segmented in each image using the Grounded Segment Anything with the prompt “face,” and face size was measured as the total number of pixels assigned to all faces within the image.

#### 2.3.3 Number of people

To define the number of people in an image, we extracted the segmentation labels provided by the Grounded Segment Anything for each image and counted the number of “person” labels.

#### 2.3.4 Distance from the center

We used the output from the Grounded Segment Anything, particularly the bounding box of the largest human body in each image. The distance was computed as the Euclidean distance between the center of an image and the center of the bounding box.

#### 2.3.5 RMS contrast

We converted each image to grayscale values and computed the RMS contrast of the pixel intensities as a coarse proxy for the spatial structure. This value tends to be lower when large uniform regions occupy the image.

### 2.4 Implied motion rating task

#### 2.4.1 Participants

One of the authors and four naive paid volunteers (mean age ± SD = 25.0 ± 1.67 years) with corrected-to-normal vision participated in the behavioral experiment. Our experiments were conducted with permission from the institutional ethics review board. Written informed consent was obtained from all the participants prior to enrollment, and the Declaration of Helsinki guidelines were followed. Ratings of one of the authors (Subject 01) were strongly correlated with those of the other four subjects (Supplementary Figs. S1B, S6B and S7B) and, therefore, did not bias the results.

#### 2.4.2 Image selection

Out of the 73,000 natural scene images included in the NSD, we sampled a subset of those images for our rating experiment. We focused on 1,000 shared images that were viewed by all 8 subjects in the NSD experiment and used the image segmentation data (specifically the “person” label) included in the original COCO category annotations to select images containing human bodies. We then selected a subset of images by visual inspection for the rating experiment according to the following two criteria, which we believe facilitate judgments of implied motion in the depicted person, without applying a fixed quantitative size threshold. First, we ensured that all images do not contain animals or vehicles that potentially imply motion information unrelated to that of the person in an image (for example, we excluded images of a person riding a horse). Second, we eliminated images in which the person appears too small in the background because inferring the implied motion of a person when the person occupies only a small portion of the image is difficult. The mean pixel proportion of the person in the excluded images was 1.2%, whereas that in the included images was 21.6%. After these 2 screening criteria were applied, 100 images were randomly selected from the resulting set. In addition to these 100 person images, we also sampled animal and vehicle images from the shared images labeled with the COCO superordinate categories “animal” and “vehicle.” We again subsampled 100 images for each category based on the same criteria as above, ensuring that all the animal (vehicle) images did not contain people or vehicles (animals) and that the animals and vehicles in all the images did not appear too small. The mean pixel proportion of the animal and vehicle in the excluded images was 0.44% (animal) and 0.13% (vehicle), whereas that in the included images was 19.2% and 19.8%, respectively. For the vehicle category, only 88 images met the criteria. Consequently, a total of 288 natural images were selected to conduct an implied motion rating task for 3 categories.

#### 2.4.3 Procedure

Our rating experiment consisted of three sessions in total, one for each target category (person, animal, and vehicle), and the order of these sessions was counterbalanced across participants. Each session started with a passive-viewing session in which participants observed all images of a target category, each presented for 1 second, without response, so that they were aware of the dynamic range of motion information implied in the images (Lu et al., 2016), making it easier for participants to rate the implied motion speed of a target category in subsequent rating blocks. In the next four blocks, participants were presented with an image for 3 seconds and evaluated the speed of implied motion of a target category for each image on a scale of 1 to 5. We provided participants with clear rating criteria: 1 indicates no motion and 5 corresponds to the fastest motion they had previously seen in the passive-viewing block. We also instructed participants to give a rating within 3 seconds of image presentation by considering the overall scene context in which the target category is present rather than relying solely on the semantic category of an image (e.g., we expected them to assign different ratings to an image of a surfer riding a wave and an image of a surfer just standing on the shore). Each image was presented twice throughout the 4 blocks; thus, the participants completed a total of 200 trials for the person and animal categories (50 trials per block) and 176 trials for the vehicle category (44 trials per block), with a few minutes of rest after 2 blocks. Prior to these main blocks, we also conducted a practice block in which participants rated the implied motion speed of 20 NSD images for each target category that were not included in the 288 images used in the main experiment based on the same criteria as above. Each image was presented on a monitor (BenQ XL2546X-B) with a size of 8.4° × 8.4°, at a viewing distance of 50 cm, followed by a 1-second blank gray background, in accordance with the conditions of the NSD experiment (Allen et al., 2022).

#### 2.4.4 Rating reliability

We assessed two types of reliability for the ratings from our task: within-participant reliability, computed as Spearman’s rank correlation between ratings from the first and second image presentations for each participant, and between-participant reliability, computed as Spearman’s rank correlation between ratings averaged across two repetitions for each pair of participants. We confirmed high within-participant and between-participant reliability (Supplementary Figs. S1, S6, and S7). Thus, we averaged the ratings across repetitions and participants and used these average ratings for subsequent analyses.

### 2.5 Variance partitioning

To examine the unique and shared contributions of three major body-related features (implied motion, body size, and number of people) to the prediction of brain responses for each vertex, we performed variance partitioning based on seven linear regression models fitted using ordinary least squares. Specifically, we quantified the amount of variance uniquely explained by each feature, as well as the variance jointly explained by two or all three features. Explained variance was quantified using the coefficient of determination (R²), which was computed for each regression model via five-fold cross-validation. For each fold, the linear regression model was trained on 80% of the data and tested on the remaining 20%. The cross-validated R² values obtained from the test sets were averaged across the five folds to robustly estimate the model performance. On the basis of the seven cross-validated R² values ($R_{i}^{2},\;R_{n}^{2},\;R_{s}^{2},\;R_{is}^{2},\;R_{in}^{2},\;R_{ns}^{2},\;R_{ins}^{2}$ for implied motion, number of people, body size, a pair of implied motion and body size, a pair of implied motion and number of people, a pair of body size and number of people, and a combination of all three features, respectively), we computed the unique variance for each feature and the shared variance for all possible combinations of features. For instance, the uniquely explained variance for implied motion ($U_{i}$) was computed as follows:

$$U_{i}=R_{ins}^{2}-R_{ns}^{2}.$$

The variance shared by the implied motion and number of people ($S_{in}$ ) and among all three features ($S_{ins}$ ) was computed via the following equations:

$$S_{in}=R_{is}^{2}+R_{ns}^{2}-(R_{s}^{2}+R_{ins}^{2}).$$

$$S_{ins}=R_{ins}^{2}-\left(R_{in}^{2}+R_{is}^{2}+R_{ns}^{2}\right)+\left(R_{i}^{2}+R_{n}^{2}+R_{s}^{2}\right).$$

Statistical significance was assessed using a permutation test as described above. We used the same randomization for all seven regression models and evaluated the significance of the observed cross-validated R² values based on the significance threshold (*p* < 0.05; FDR-corrected) defined by a null distribution of R² values for each vertex. We then extracted the vertices for which at least one of the seven regression models achieved significant prediction accuracy and computed the proportion of vertices that were best explained by each feature or a combination of multiple features.

## 3 Results

### 3.1 Data-driven analysis of object co-occurrence revealed object pairs associated with the EBA response

The present study aimed to understand precisely what aspects of natural scenes contribute to semantic representations in the body-selective region that are captured by LLM embeddings of natural scene captions (Doerig et al., 2025; Horikawa, 2025). To this end, we first constructed caption-based encoding models following these previous studies as a preparatory step. We then developed an analysis of the relationships between the co-occurrence patterns of objects in large-scale natural scene images and the corresponding EBA responses predicted by the encoding models (Fig. 1), motivated by the idea that the joint presence of multiple objects generally shapes the overall semantic content of a scene. Through this analysis, we sought to reveal which pairs of object categories are associated with the magnitude of the EBA response and to characterize the semantic information represented in the EBA in an interpretable manner.

Our co-occurrence analysis framework is centered around a co-occurrence matrix. Each element in this matrix reflects the co-occurrence frequency of words of multiple object categories in a set of captions that provide textual descriptions of natural scenes (see Fig. 1A as an example). We used 12 MS COCO superordinate object categories: accessory, animal, appliance, electronic, food, furniture, indoor, kitchen, outdoor, person, sports, and vehicle (see Supplementary Table S1 for subordinate categories for each of the 12 categories). The matrix captures important information regarding the co-occurrence patterns in a set of captions. One rationale for using image captions instead of the objects depicted in the images themselves is that images often contain too many objects, some of which may be irrelevant to the overall semantic content of a scene (e.g., a small cup on a desk when people have a meeting in a room). In contrast, captions capture the essential components of a scene, providing information that aligns closely with human semantic scene recognition.

Our co-occurrence analysis involved the following steps. First, we trained encoding models that predict mean EBA responses for each subject in the NSD (Allen et al., 2022), using embeddings derived from human-annotated captions of the scene images (Fig. 1B). Next, we used these trained models to predict EBA responses to all 73,000 captions and divided these captions into 73 groups of 1,000 captions in descending order of the average predicted EBA responses. For each group, we constructed a co-occurrence matrix (Fig. 1C). We subsequently performed nonnegative matrix factorization to extract major co-occurrence patterns that are predominantly represented in the 73 matrices and their contributions to the original matrix of each caption group. By using predicted responses from the encoding models, we leveraged the entire set of 73,000 caption–response pairs in the NSD, which far exceeds the 1,000 observed responses shared across 8 subjects.

**Fig. 2.**
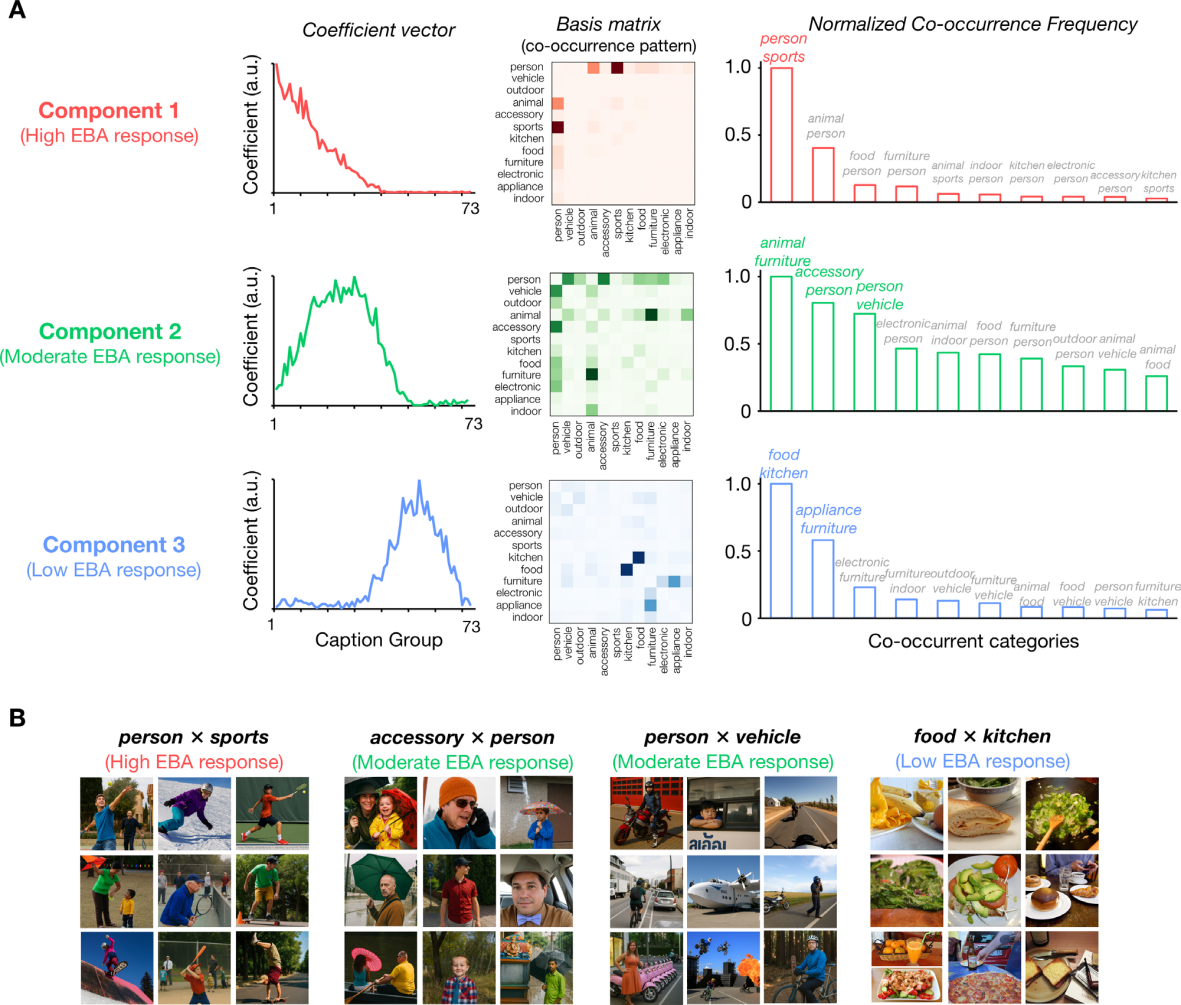
Results of the co-occurrence analysis. (A) Three co-occurrence components were revealed by non-negative matrix factorization applied to the 73 co-occurrence matrices. Each component is associated with high, moderate, and low EBA responses, respectively, as indicated by the different peak locations of the coefficient vectors along the horizontal axis (left), which represents the caption groups sorted by predicted EBA response magnitudes. The middle panels show the co-occurrence patterns for the three components, which are summarized as the bar plots of normalized co-occurrence frequency of the top 10 co-occurring category pairs (right). The pair labels are colored if the corresponding value exceeds 0.5. The category “person” co-occurred with “sports” in component 1, and with “accessory” and “vehicle” in component 2, suggesting that EBA responses vary depending on the object categories co-occurring with human bodies. (B) Example COCO images containing the major co-occurring category pairs. For copyright reasons, COCO images containing human faces have been replaced with images generated by ChatGPT-5, which preserve the semantic content and composition of the original images.

The caption-based encoding models showed a high capacity to predict the overall EBA response for all the subjects (Pearson correlation ranging from 0.44 to 0.72). Based on the predicted responses from these high-performing encoding models, the co-occurrence matrices for 73 groups of captions were decomposed into 3 components (hereinafter, components 1 to 3), each associated with high, moderate, and low EBA responses, as shown by the distinct peak locations of the corresponding coefficient vectors (Fig. 2A). The bar plots in Figure 2A show the top 10 category pairs ranked by co-occurrence frequency for each component. As expected, components 1 and 2 (associated with high and moderate EBA response) included the “person” category, whereas it was absent from most category pairs in component 3 (related to low EBA response). The “animal” category frequently occurred in component 2 (moderate EBA response), which is consistent with previous findings that EBA shows weaker responses to animal bodies than to human bodies (Downing et al., 2001). These results support the validity of our method for revealing the functional properties of the body-selective regions.

Notably, the “person” category co-occurred with distinct categories across components: with “sports” in component 1 and with “accessory” and “vehicle” in component 2, indicating that images of people co-occurring with sports-related equipment drive stronger responses in the EBA than those with an accessory or vehicle. These results suggest that EBA responses vary depending on the object category that co-occurs with people in a scene.

### 3.2 Extracting features that potentially constitute semantic representations in the EBA

Next, guided by the dominant co-occurrence patterns revealed by the matrix factorization and visual inspection of representative images (Fig. 2B), we identified a set of body-related features that could plausibly account for the EBA responses. We first noticed that images driving higher EBA responses tend to imply more dynamic human body motion; for example, a person hitting a ball with a racket or doing acrobatics moves faster than a person standing with an umbrella. Another aspect potentially linked to EBA responses is the number of people in the images, as playing sports often involves more than one person. Indeed, Figure 2B shows that some person–sports images contain a few people in the background, while person–accessory and person–vehicle images do not. Thus, we hypothesized that implied body motion speed and the number of people depicted in static images were potential determinants of EBA responses.

Our qualitative assessment of the images in Figure 2B also revealed several low- and mid-level image features that may covary with the co-occurring object categories. First, many of the images driving high EBA responses tend to contain relatively large human bodies in the center, while those driving moderate EBA responses tend to contain smaller forms of human bodies in the periphery. Second, some of the person–accessory images are covered by a close-up of the human face, which is not included in the person–sports images. Third, person–sports images often contain large uniform background areas (e.g., blue sky, snow field, or tennis court), resulting in lower spatial frequency content than other images. These observations raise the possibility that EBA responses can be modulated by body size, face size, distance between the image center and human body, or spatial frequency content.

Having identified six features (implied motion, number of people, body size, face size, distance from the image center, and spatial frequency) that may potentially modulate EBA responses, we extracted these features from a set of NSD images for subsequent neural correlation analyses. To define the speed of implied motion (Freyd, 1983; Kourtzi, 2004) for each image, we conducted a behavioral experiment to collect subjective ratings of implied motion speed. Five participants were presented with 100 NSD images containing human bodies and rated the implied motion of the person depicted in the image on a scale from 1 to 5. We obtained the mean ratings of the implied body motion across participants for each image (Supplementary Fig. S1A) and used them in subsequent analyses, because between-participant reliability was significantly high across all participant pairs (Spearman’s rank correlation ranged from 0.80 to 0.90, Supplementary Fig. S1B).

To compute four body-related features (body size, face size, number of people, and distance between the image center and human body), we used a text-based segmentation model (Supplementary Fig. S2A). This model receives an image and a word of object category as input and outputs segmented areas and bounding boxes (rectangular frames surrounding the segmented bodies), indicating the presence of the object in the image. For each of the 100 NSD images used in the behavioral experiment, we extracted segmented areas of people and computed the 4 body-related feature values. Additionally, the RMS contrast was computed for each image as a low-level image statistic reflecting overall luminance variability and serving as a coarse proxy for spatial frequency content.

### 3.3 Semantic representations in the body-selective regions are composed of multiple body-related features

**Fig. 3.**
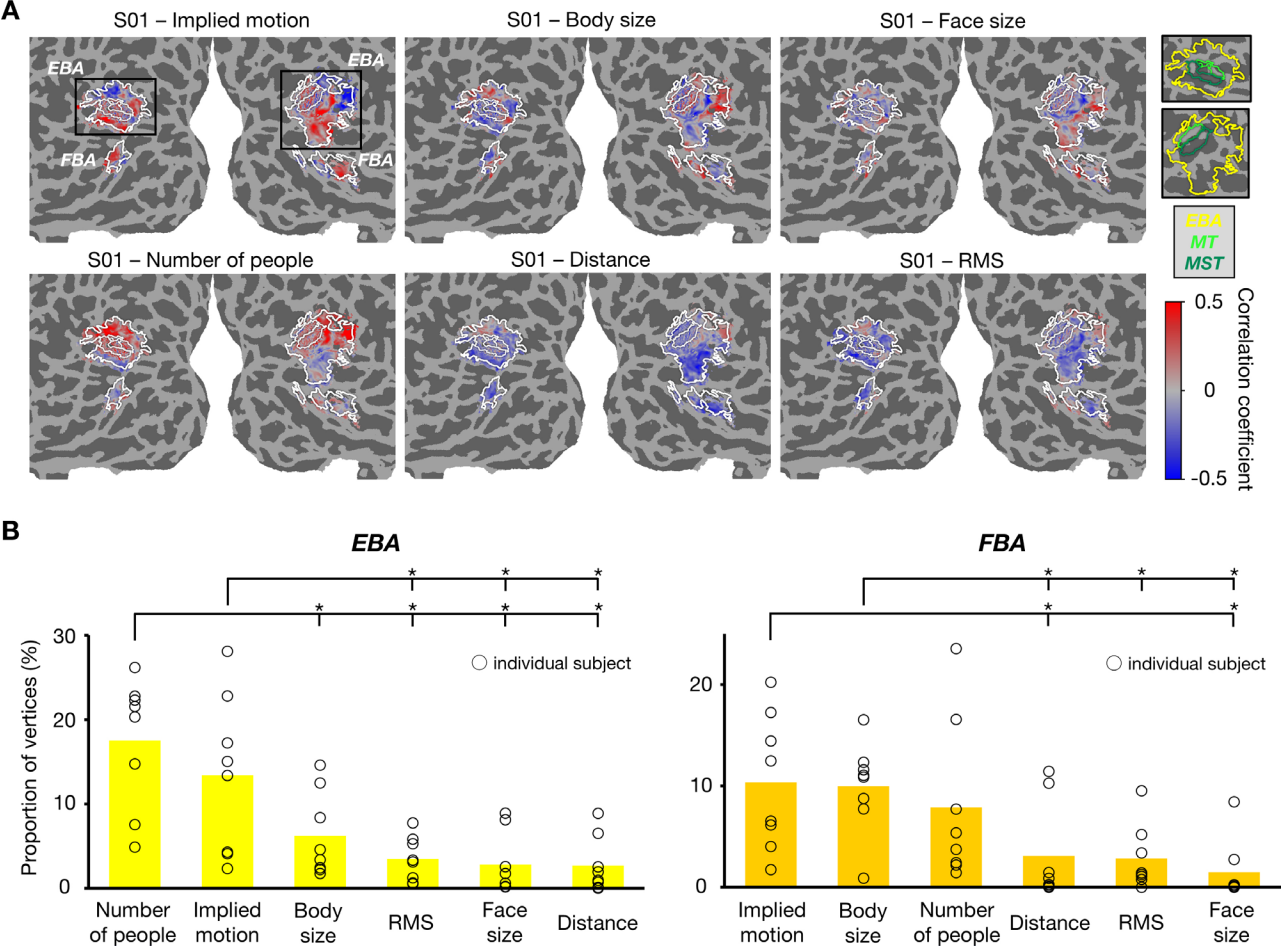
Correlation coefficients with body-related features across vertices. (A) Flattened cortical surface maps of correlation coefficients with the six body-related features for subject 01. The insets in the top right indicate the locations of overlapping regions: the EBA (yellow) and motion-selective regions (light and dark green; MT and MST). Some vertices exhibited high correlations with implied motion, body size, and number of people, whereas face size, distance from the center, and RMS contrast explained little of the responses for most vertices. (B) Proportion of vertices in the EBA and FBA showing significant correlations for each feature. The features are sorted in descending order of the average proportion. Vertices in EBA that belonged to MT or MST were excluded when computing the proportion. The significance of correlations was assessed by one-sided permutation tests with 1,000 randomizations of the stimulus labels (FDR corrected *p* < 0.05). Asterisks indicate significant differences in proportions, assessed by Wilcoxon signed-rank tests with FDR correction (*p* < 0.05). Only the pairwise comparisons indicated by brackets reached statistical significance; none of the other comparisons were significant.

Next, we assessed how the six image features extracted from the NSD images are correlated with EBA responses. After defining the EBA for each NSD subject via cortical surface t-statistic maps derived from functional localizer experiments in the NSD, we computed the correlation between the six image features and responses for each vertex. We also defined the FBA (Peelen & Downing, 2005), which is in the fusiform gyrus, to investigate whether these two anatomically separable body-selective regions differ in the representational strength of the six features. Figure 3A shows cortical maps of the correlations with the six features for one representative NSD subject (the correlation maps for the other seven subjects are provided in Supplementary Fig. S3). Several small clusters of vertices showing high positive correlations with implied motion, number of people, and body size were distributed within both the EBA and FBA. For the remaining features, although weak negative correlations with distance from the center were observed in some vertices, neither EBA nor FBA exhibited strong correlations with face size and RMS.

To quantitatively compare the spatial extent over which each feature is represented in the two body-selective regions, we performed a permutation test to compute the proportion of vertices showing significant correlations in each region. Before computing the proportions, we restricted the analysis to EBA vertices located outside the MT and MST that overlap with EBA (see light and dark green contours in Fig. 3A) (Ferri et al., 2013; Peelen et al., 2006; Weiner & Grill-Spector, 2011). This exclusion was motivated by evidence that these motion-processing regions encode implied body motion speed in natural scenes (Kourtzi & Kanwisher, 2000; Lu et al., 2016; Senior et al., 2000, 2002), which was also evident in our data (Supplementary Fig. S4) and could, therefore, confound the contribution of implied motion intrinsic to the EBA. Figure 3B shows the results of EBA (outside the MT and MST) and FBA vertices showing significant correlations with the six features, which are sorted in descending order of the average proportion. We found a significant effect of feature type for both regions (Friedman’s test, $\chi^{2}$ = 22.64, *p* < 0.001 for EBA; $\chi^{2}$ = 21.20, *p* < 0.001 for FBA). For pairwise comparisons, post hoc Wilcoxon signed-rank tests with FDR correction were performed. For EBA, the largest proportions of significantly correlated vertices were observed for implied motion and number of people. Number of people showed higher proportions than body size (*T* = 0.0, *p* = 0.029), RMS (*T* = 1.0, *p* = 0.034), face size (*T* = 1.0, *p* = 0.034), distance (*T* = 0.0, *p* = 0.029) but did not differ from implied motion (*T* = 8.0, *p* = 0.29). Implied motion showed higher proportions than RMS (*T* = 1.0, *p* = 0.034), face size (*T* = 0.0, *p* = 0.029), and distance (*T* = 0.0, *p* = 0.029), but not body size (*T* = 3.0, *p* = 0.065). For FBA, the largest proportions of significantly correlated vertices were observed for implied motion and body size. Implied motion showed higher proportions with distance (*T* = 0.0, *p* = 0.029) and face size (*T* = 0.0, *p* = 0.029), but did not differ from body size (*T* = 18.0, *p* = 1.0), number of people (*T* = 15.0, *p* = 0.86), and RMS (*T* = 2.0, *p* = 0.059). Body size showed higher proportions than distance (*T* = 1.0, *p* = 0.047), RMS (*T* = 0.0, *p* = 0.029), and face size (*T* = 0.0, *p* = 0.029). All other pairwise comparisons were not statistically significant (*p* > 0.05). Taken together, implied motion emerged as the most broadly represented feature across both regions, whereas number of people and body size contributed to both regions but were relatively more prominent in the EBA and FBA, respectively, indicating that these three features dominate semantic representations in body-selective regions.

### 3.4 Variance partitioning identified the largest functional cluster uniquely explained by implied motion

Our results suggest that responses in body-selective regions can be captured by multiple body-related features, especially implied body motion, body size, and the number of people in natural scenes. This leads to the following question: to what extent are such features uniquely represented within these regions? As the body-related features are not orthogonal to each other (a correlation matrix of all pairs of features is provided in Supplementary Figure S2B), simply constructing cortical correlation maps and computing the proportion of vertices with significant correlations for each feature is insufficient to reveal the fine-grained cortical organization of features uniquely encoded in the body-selective regions. A more appropriate approach is to quantify the amount of response variance uniquely explained by each individual feature. Thus, we performed variance partitioning (de Heer et al., 2017; Lescroart et al., 2015) using the three features most strongly associated with EBA and FBA—implied motion, number of people, and body size—and identified cortical locations where each of these features best accounted for responses. We fitted seven linear regression models separately, each incorporating either a single feature or a combination of two or three features, and computed the five-fold cross-validated R-squared values for each model to quantify the variance uniquely explained by individual features and the shared variance jointly explained by feature pairs or triplets (see Methods for details).

**Fig. 4.**
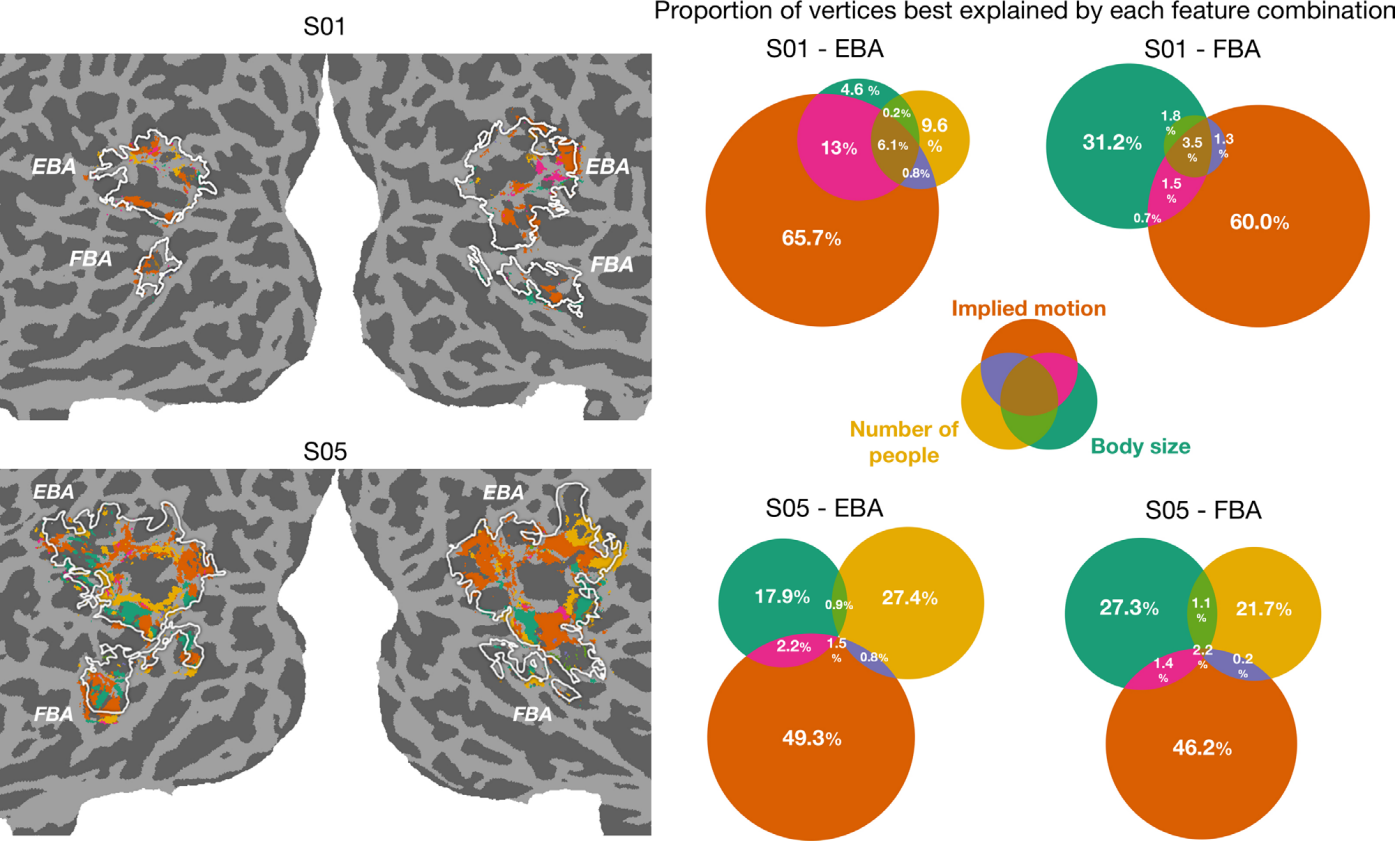
Cortical surface maps of the largest variance partition. Each vertex is assigned a color corresponding to the body-related feature that explains the largest fraction of variance for two representative subjects (subjects 01 and 05). Vertices are not assigned a color if they are also labeled as MT or MST, or if none of the unique and shared variances are significant. The Venn diagrams summarize the proportion of vertices best explained by each partition (note that the size of each area is not scaled to reflect exact proportions).

We constructed cortical maps indicating the largest variance partition for each vertex (Fig. 4). For example, vertices shown in orange exhibited the largest variance uniquely explained by implied motion, and vertices shown in magenta exhibited the largest variance jointly explained by implied motion and body size. To quantify the size of each colored cluster, we then computed the proportion of vertices for which each variance partition was maximal (Fig. 4; see Supplementary Fig. S5 for all subjects). We found that most vertices were best and uniquely explained by one of the three features alone, with only a small portion of the vertices jointly explained by two or three features, as indicated by the small intersections of the Venn diagrams. Notably, implied body motion best explained the largest fraction of vertices in the EBA for seven out of eight subjects and in the FBA for five out of eight subjects. For half of the subjects, the second largest proportion of vertices in the EBA was best explained by the number of people (subjects 2, 4, 5, and 7). In contrast, body size best accounted for the second largest proportion in the FBA (subjects 1, 2, 3, 5, and 7). These results suggest that multiple aspects of human bodies in natural scenes jointly contribute to the representation in body-selective regions in a complementary manner, with implied body motion serving as the primary contributor.

### 3.5 Body-selective regions represent implied motion specifically for human bodies, but not for non-human categories

An important question raised by the above results is whether the representation of implied motion in body-selective regions reflects a category-general sensitivity to implied motion or a category-specific encoding tied to human bodies. Given that the EBA is anatomically proximal to motion-processing regions (MT and MST), the EBA may encode implied motion in a category-general manner, such that implied motion is represented for both human bodies and other categories. To test this hypothesis and gain additional insights into the content of semantic representations in the body-selective regions, we conducted the same rating experiment using NSD images of animals and vehicles to collect implied motion ratings for these categories. Again, we obtained one average rating across subjects for each image (Supplementary Figs. S6A and S7A) and used it for subsequent correlation analysis because of the significantly high between-participant reliability (Spearman’s rank correlation ranging from 0.40 to 0.71 for the animal images and 0.81 to 0.90 for the vehicle images; Supplementary Figs. S6B and S7B).

**Fig. 5.**
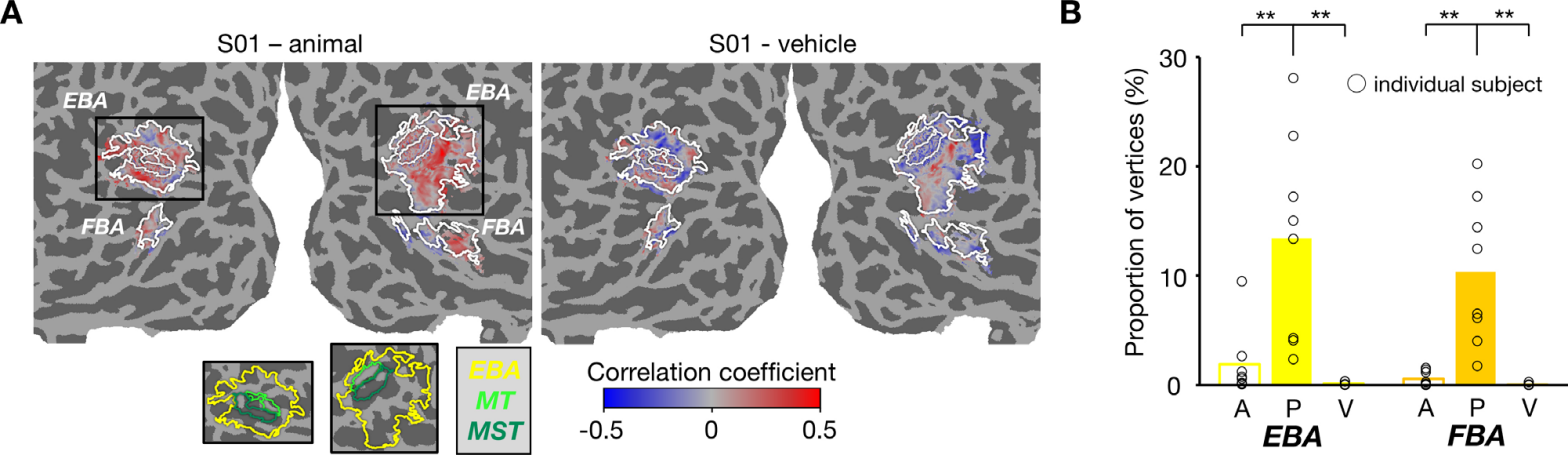
Cortical correlation maps for implied motion of animals and vehicles. (A) Correlation maps for animal and vehicle images from one NSD subject (subject 01). The insets at the bottom indicate the locations of overlapping regions: the EBA (yellow) and motion-selective regions (light and dark green; MT and MST). A small portion of vertices in EBA exhibits slightly positive correlations with animal implied motion, but not with vehicle implied motion. (B) Proportion of vertices showing significant correlation with implied motion for animal (A), person (P), and vehicle (V) images within body-selective regions. Again, the EBA vertices included in the MT or MST were excluded to compute the proportion. The significance of vertex-wise correlations was assessed by one-sided permutation tests, with 1,000 randomizations of the stimulus labels (FDR corrected *p* < 0.05). Asterisks indicate significant differences in proportions determined by Wilcoxon signed-rank tests with FDR correction (**: *p* < 0.01).

Figure 5A shows cortical maps of the correlations between the brain responses and implied motion ratings of animals and vehicles for one representative subject (correlation maps for all subjects are provided in Supplementary Fig. S8). A small portion of vertices showed slightly positive correlations with implied motion for animal images; however, in contrast to the correlation maps for implied motion of person images (Fig. 3A), these correlation maps, particularly for vehicles, did not show strong correlations in most vertices. We again assessed the significance of vertex-wise correlations for each category and computed the proportion of vertices with significant correlations within each body-selective region (Fig. 5B). The proportion of vertices significantly correlated with the implied motion for the animal and vehicle images was substantially smaller than that for the person images (Wilcoxon signed-rank tests, *T* = 0.0, *p* = 0.007). These results indicate that although humans perceive implied motion across multiple categories, the EBA and FBA selectively represent implied motion for human bodies, with only weak representations for non-human categories.

## 4 Discussion

The primary goal of this study is to understand which aspects of natural scenes are particularly important for driving EBA responses and to better characterize the semantic representations in the EBA that were captured by embeddings of natural scene captions (Doerig et al., 2025; Horikawa, 2025). Using caption-based encoding models as a starting point, we first developed an analysis focusing on the relationship between the co-occurrence patterns of multiple objects in a wide array of natural scene images and the corresponding predicted brain responses. This analysis revealed several category pairs that are associated with the magnitude of the EBA response and identified six image features that may potentially modulate EBA responses. Subsequent correlation analyses showed that a large portion of EBA and FBA vertices exhibited significant correlations with three of the six features: number of people, body size, and the speed of implied human body motion that was evaluated in our behavioral experiment. We also disentangled the contributions of these three body-related features to the semantic representations in EBA and FBA and found that implied motion, number of people, and body size depicted in scene images uniquely explain responses in distinct cortical locations of the EBA and FBA, with the largest cluster associated with implied human body motion. Our framework, which combines co-occurrence analysis with caption-based encoding models, enabled us to extend beyond the traditional view of body selectivity and interpret what contributes to semantic representations in body-selective regions.

Several studies have examined neural responses in high-level visual regions to object pairs (Cox et al., 2004; Harel et al., 2013; Kim & Biederman, 2011; Zoccolan et al., 2005) and proposed a computational framework to explain those responses (Bao & Tsao, 2018; Doostani et al., 2023; Kliger & Yovel, 2020). However, these efforts were constrained by using a handful of object pairs that were manually selected from many possible pairs. Another line of research has leveraged the statistical structure of object co-occurrence in natural scenes to generate embeddings for predicting brain responses (Bonner & Epstein, 2021; Stansbury et al., 2013). Although these embeddings capture neural representations of object and scene perception, this approach does not identify the specific object pairs leading to such responses. Our co-occurrence analysis examined the co-occurrence frequencies across 66 pairs of object categories across 73,000 natural scene captions and extracted, in a data-driven manner, co-occurrence patterns associated with responses of the body-selective region predicted by caption-based encoding models. As a result, we identified the object categories co-occurring with human bodies that determine the magnitude of responses in the body-selective region: sports driving the strongest responses, and accessories and vehicles driving moderate responses. Moreover, qualitative assessment of images containing these co-occurring categories revealed several body-related image features that may explain responses in the EBA, three of which exhibit strong correlations across a large portion of the EBA.

To directly evaluate the effect of the large sample size used to derive the co-occurrence patterns, we additionally performed the co-occurrence analysis using only the actual EBA responses to the 1,000 images shared across subjects, with 10 co-occurrence matrices defined from 10 groups of 100 captions. We found that the BIC was minimized by a single component, failing to extract even two distinct components corresponding to person and non-person categories, which are associated with strong and weak EBA responses, respectively, despite the well-established body selectivity of the EBA for human bodies. Therefore, leveraging the full set of 73,000 caption–response pairs through encoding model predictions, rather than measured responses to only a subset of those images, is crucial for reliably identifying object co-occurrence patterns and the three body-related features associated with EBA responses. In the following paragraphs, we discuss each of the three features (implied motion, number of people, and body size) in detail.

Previous fMRI studies have shown that activity in the EBA is suppressed during repeated presentation of identical actions in short video clips, indicating that the EBA represents the category of body actions (Kable & Chatterjee, 2006; Takahashi et al., 2008; Wiggett & Downing, 2011). We extend these findings by showing that the EBA encodes the category of actions inferred from co-occurring objects in static images (i.e., sports-related versus accessories and vehicles) and represents the speed of actions, even in the absence of real motion. Moreover, the FBA—anatomically distinct from both the motion-selective areas and EBA—also showed strong correlations with the speed of implied motion. This highlights a new aspect of FBA involvement in processing implied body motion. Considering these findings, the representation of implied motion, previously reported in motion-selective regions (Kourtzi & Kanwisher, 2000; Lu et al., 2016; Senior et al., 2000, 2002), is more widely distributed in the brain and points to a broader functional role of the EBA and FBA in representing human body motion inferred from accompanying objects and bodies themselves, rather than merely responding to the presence of human bodies in natural scenes.

Although variance partitioning showed that implied motion uniquely and best explained the responses of the largest proportion of vertices in the EBA and FBA for most subjects, the number of people depicted in the images also uniquely explained the responses in distinct subregions of these areas. This finding mirrors results from recent studies identifying two clusters within the EBA, each representing either individual bodies or bodies of multiple people (Abassi & Papeo, 2020; Luo et al., 2024). However, the clusters reported in these studies encompassed almost the entire EBA—an apparent overestimation of the extent of the cluster reflecting the number of people—because the contributions of multiple body-related features were not disentangled in these studies. Another finding from variance partitioning was that the EBA and FBA differ in the second largest contributing body-related feature: the number of people for the EBA and the body size for the FBA. This regional difference is in line with previous studies showing that these two regions differ in several dimensions (Abassi & Papeo, 2020; Foster et al., 2019; Hodzic et al., 2009; J. Ren et al., 2022; van de Riet et al., 2009). We speculate that the difference observed in our study stems partly from distinct patterns of structural and functional connectivity between these regions: the EBA connects primarily with the superior parietal lobe in the dorsal stream, whereas the FBA shows strong connectivity with the fusiform gyrus and inferior temporal cortex in the ventral stream (Zimmermann et al., 2018). As the superior parietal lobe plays a role in encoding object locations (Colby & Goldberg, 1999; Goodale & Milner, 1992) and shifting spatial attention (Corbetta et al., 1995; Vandenberghe et al., 2001), which may be necessary to count the number of people distributed over an image, the EBA is more likely to represent that information. Conversely, estimating body size requires shape processing of these bodies, which is supported by regions in the ventral stream (Kravitz et al., 2013; Logothetis & Sheinberg, 1996), thus resulting in a pronounced representation of body size in the FBA. Collectively, the EBA and FBA responses are modulated by several body-related features whose relative importance varies across regions, in part due to the distinct patterns of connectivity.

Recent theoretical accounts propose that the EBA is located at the intermediate stage of a visual pathway along the lateral brain surface dedicated to processing dynamic actions relevant to social interactions (Pitcher & Ungerleider, 2021; Wurm & Caramazza, 2022). The three body-related features identified in our analysis are indeed crucial for understanding others’ actions and guiding interactions with them; for example, the number of people conveys information regarding the social context of an action, and body size enables estimation of the physical proximity of another person to one’s body. Based on these observations, we speculate about the general computational roles of the EBA: it may integrate low- to mid-level information, such as orientation and shape, derived from early visual regions to compute several body-related features, including the three interpretable features identified here as well as additional features that remain difficult to interpret (Frisby et al., 2023). These body-related features may comprise semantic representations in the EBA and serve as intermediate building blocks that are relayed to downstream regions, leading to accurate action recognition.

A significant gap remains regarding a comprehensive understanding of the semantic representations in body-selective regions, as indicated by the fact that our linear regression model incorporating the three body-related features (implied motion, number of people, and body size) did not successfully explain all vertices. This is natural as the features derived from the NSD images do not fully capture all aspects of human bodies in natural scenes. For example, emotion and social information expressed in the human body, which was previously reported to influence responses in the EBA and FBA (Marrazzo et al., 2021; Marsh et al., 2010; McMahon et al., 2023; Poyo Solanas et al., 2020a), may be a key factor that potentially improves the predictability of these regions. As deriving emotion-related features from NSD images that mostly contain emotionally neutral and nonsocial scenes is inherently difficult, future studies should employ diverse sets of natural images and construct a model incorporating affective (Abdel-Ghaffar et al., 2024), social (Gandolfo et al., 2024; McMahon et al., 2023), and postural features associated with emotion categories (de Gelder & Poyo Solanas, 2021; Poyo Solanas et al., 2020b), as well as the body-related features used in the present study to better interpret the semantic content represented in body-selective regions. Additionally, while most of our analyses were conducted at the single-vertex level, distributed response patterns across multiple vertices may encode body-related information, such as body posture, as shown in previous studies (Marrazzo et al., 2023; Poyo Solanas et al., 2020a; Zhu et al., 2024). Further work is necessary to understand how information at different spatial scales is integrated to form semantic representations and achieve stable scene recognition.

Overall, we proposed a co-occurrence analysis framework that advances the traditional view of category selectivity but still preserves the interpretability of neural representations in a target region. We believe that this framework contributes to the field of cognitive neuroscience, as recent methodological advances have made it increasingly challenging to interpret results owing to the complexity of features used to model brain responses. This framework was demonstrated to be effective at elucidating the contents of semantic representations in body-selective regions, which comprise several interpretable body-related features. Future studies could apply our framework to other category-selective regions and extend it to a broader range of categories to gain a deeper understanding of high-level semantic representations in the brain.

## Supplementary Material

Supplementary Material

## Data Availability

The Natural Scenes Dataset is publicly available at http://naturalscenesdataset.org. The behavioral data and code used for the analyses reported here are available on Figshare at https://doi.org/10.6084/m9.figshare.32348043 and on GitHub at https://github.com/amano-k-lab/interpret_semrep.
