# Supplementary material for: Probing the content of semantic representations in body-selective regions
Source: Imaging Neurosci (Camb). 2026 Jul 27;4:IMAG.a.1309. doi: 10.1162/IMAG.a.1309 (PMC13409281; doi:10.1162/IMAG.a.1309)
Supplement: Supplementary Material [file IMAG.a.1309_supp.pdf]

Supplementary Materials

accessory	***animal***	***appliance***	***electronic***	***food***	***furniture***
backpack	bear	blender	cell phone	apple	bed
eye glasses	bird	microwave	keyboard	banana	chair
handbag	cat	oven	laptop	broccoli	couch
hat	cow	refrigerator	mouse	cake	desk
shoe	dog	sink	remote	carrot	dining table
suitcase	elephant	toaster	tv	donut	door
tie	giraffe			hot dog	mirror
umbrella	horse			orange	potted plant
	sheep			pizza	toilet
	zebra			sandwich	window

indoor	***kitchen***	***outdoor***	***person***	***sports***	***vehicle***
book	bottle	bench	baby	baseball	airplane
clock	bowl	fire hydrant	boy	bat	bicycle
hair brush	cup	parking meter	child	ball	boat
hair drier	fork	stop sign	girl	frisbee	bus
scissors	knife	street sign	man	glove	car
teddy bear	plate	traffic light	person	kite	motorcycle
toothbrush	spoon		woman	racket	train
vase	wine glass			skateboard	truck
				skis	
				snowboard	
				sports	
				surfboard	
				tennis	

Supplementary Table 1. List of 12 superordinate categories defined in the MS COCO dataset and 100 subordinate categories used in the co-occurrence analysis.

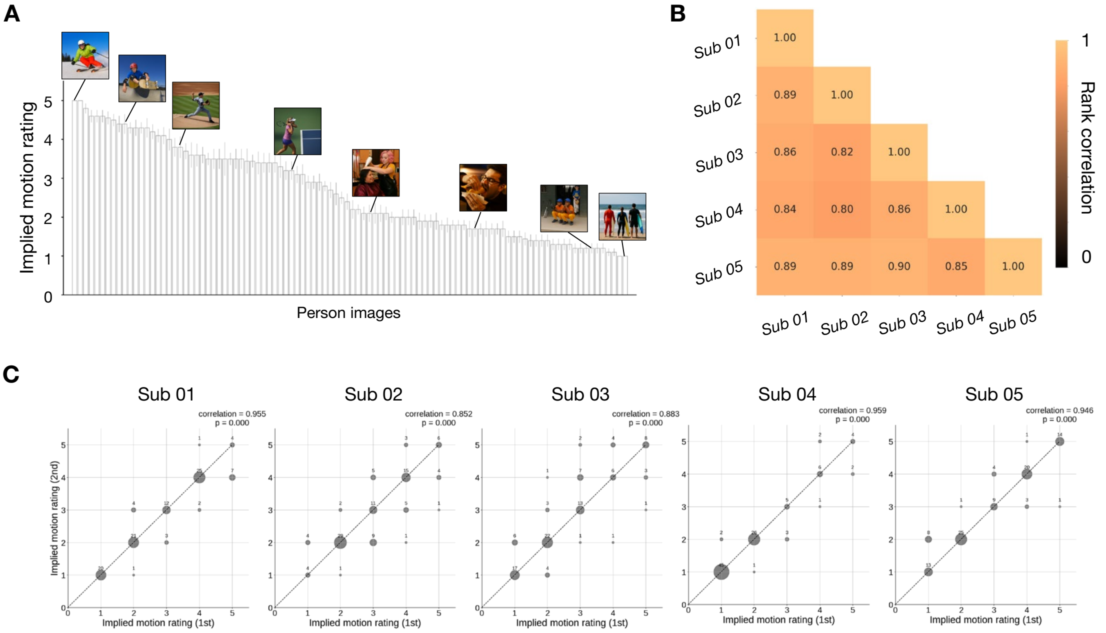

Supplementary Figure 1. Results from the implied motion rating experiment for person images. **(A)** Bar plots show ratings averaged across five participants and two repetitions for people depicted in 100 NSD images used in the behavioral experiment. Ratings were broadly distributed across the full range, from 1 (static) to 5 (fastest motion of all images). Images within the same object category (e.g., sports-related scenes) were rated as implying different levels of motion speed, indicating that participants relied on contextual information rather than category membership alone. For copyright reasons, COCO images containing human faces have been replaced with images generated by ChatGPT-5, which preserve the semantic content and composition of the original images**.** **(B)** Between-participant reliability (Spearman rank correlations of responses between all pairs of five subjects) in the rating experiment for person images. **(C)** Within-participant reliability (Spearman rank correlation of responses between the first and second image presentations) in the rating experiment for person images. Each bubble plot shows the frequency of ratings across two repetitions, with larger bubbles reflecting more frequent responses.

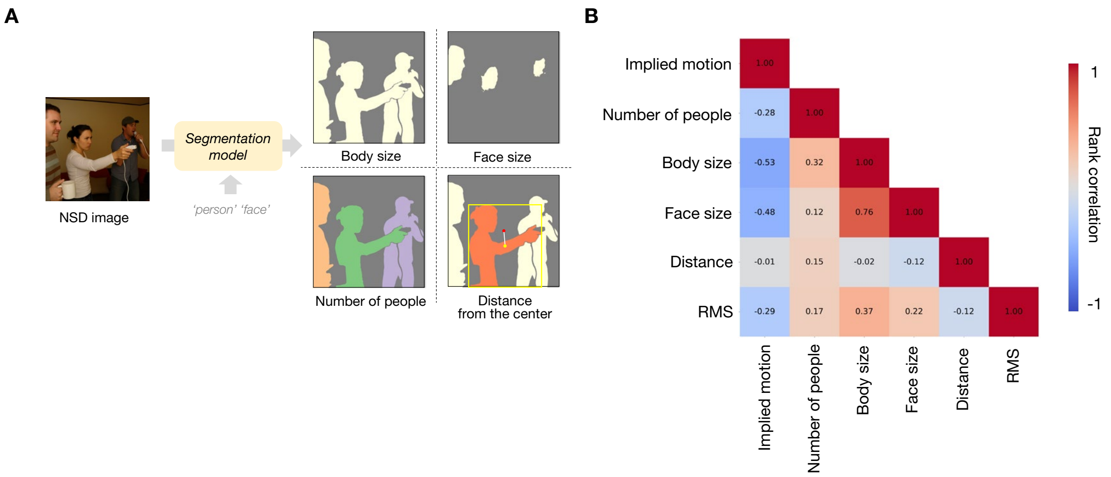

Supplementary Figure 2. (A) Examples of output from a segmentation model (Grounded Segment Anything). Text input was used to obtain images with segmented human body/face parts and bounding boxes (a yellow rectangle in the image on the right as an example). Based on these output images, we computed body size as the number of pixels corresponding to bodies, face size as the number of pixels corresponding to faces, number of people as the number of bounding boxes with 'person' labels, and distance from the center as the Euclidean distance between the center of an image and that of the largest bounding box. **(B)** Rank correlation between all pairs of features.

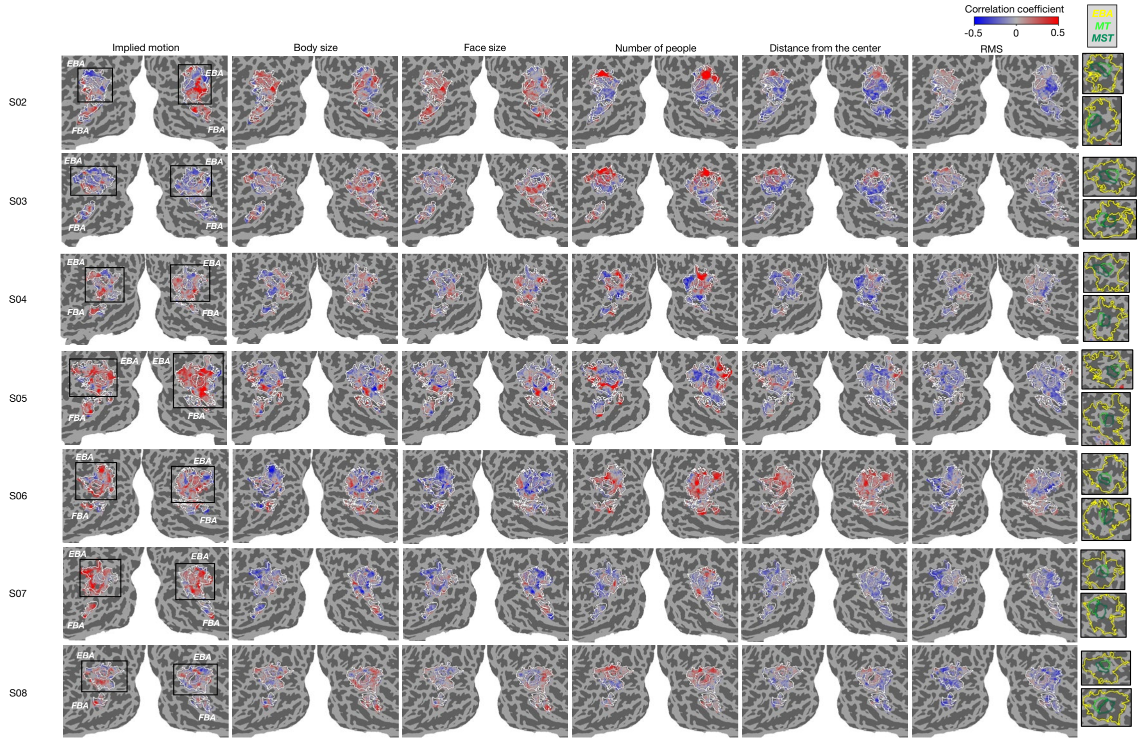

Supplementary Figure 3. Cortical surface maps of the correlation between vertex-wise responses and six features of person images for the remaining seven NSD subjects. The insets on the right indicate the locations of overlapping regions: the EBA (yellow) and motion-selective regions (light and dark green; MT and MST). The map for subject 01 is shown in Figure 3A.

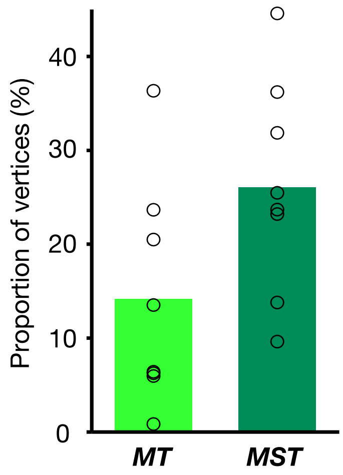

Supplementary Figure 4. Proportion of vertices showing significant correlation with implied motion for person images within the motion-selective regions.

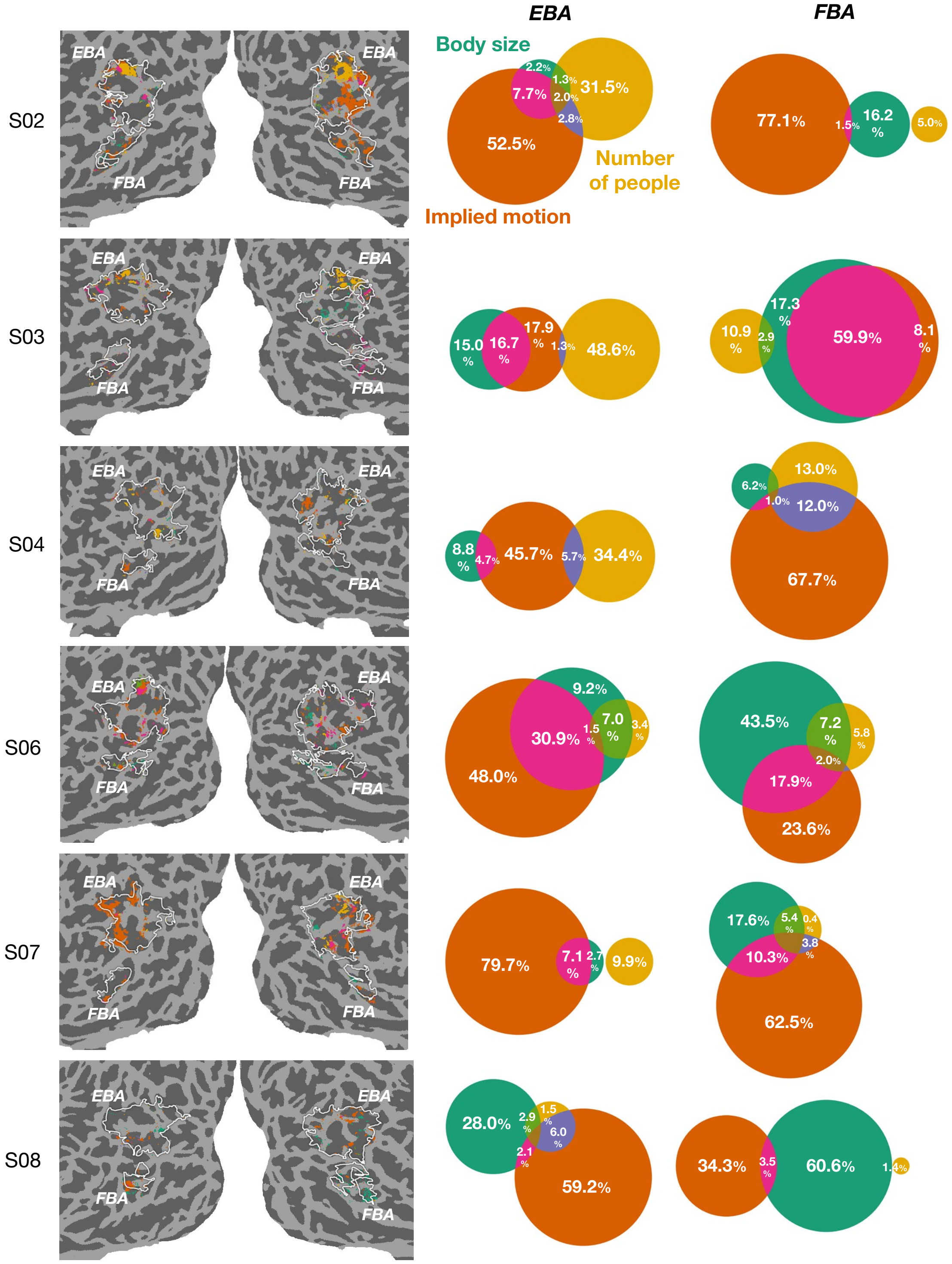

Supplementary Figure 5. Cortical surface maps of the largest variance partition for the remaining six NSD subjects. The Venn diagrams show the proportion of significantly predicted vertices best explained by each feature (note that the size of each area is not scaled to reflect exact proportions). The color conventions are the same as in Figure 4.

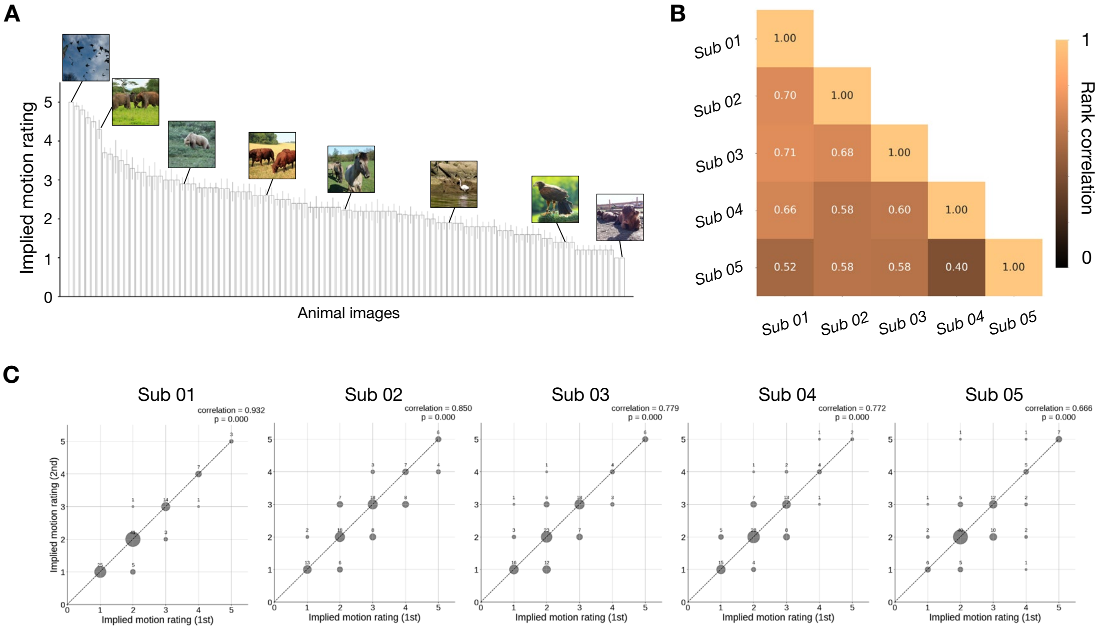

Supplementary Figure 6. Results from the implied motion rating experiment for animal images. **(A)** Bar plots show ratings averaged across five participants and two repetitions for animals depicted in 100 NSD images used in the behavioral experiment. Ratings were broadly distributed across the full range, from 1 (static) to 5 (fastest motion of all images). **(B)** Between-participant reliability (Spearman rank correlations of responses between all pairs of five subjects) in the rating experiment for animal images. **(C)** Within-participant reliability (Spearman rank correlation of responses between the first and second image presentations) in the rating experiment for animal images. Each bubble plot shows the frequency of ratings across two repetitions, with larger bubbles reflecting more frequent responses.

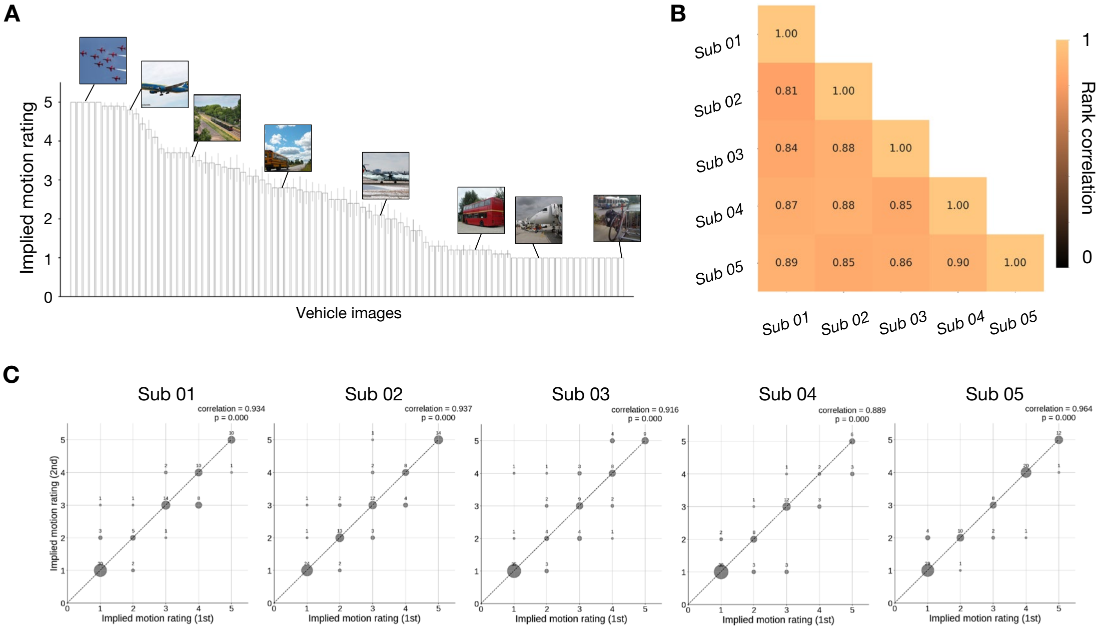

Supplementary Figure 7. Results from the implied motion rating experiment for vehicle images. **(A)** Bar plots show ratings averaged across five participants and two repetitions for vehicles depicted in 100 NSD images used in the behavioral experiment. Ratings were broadly distributed across the full range, from 1 (static) to 5 (fastest motion of all images). **(B)** Between-participant reliability (Spearman rank correlations of responses between all pairs of five subjects) in the rating experiment for vehicle images. **(C)** Within-participant reliability (Spearman rank correlation of responses between the first and second image presentations) in the rating experiment for vehicle images. Each bubble plot shows the frequency of ratings across two repetitions, with larger bubbles reflecting more frequent responses.

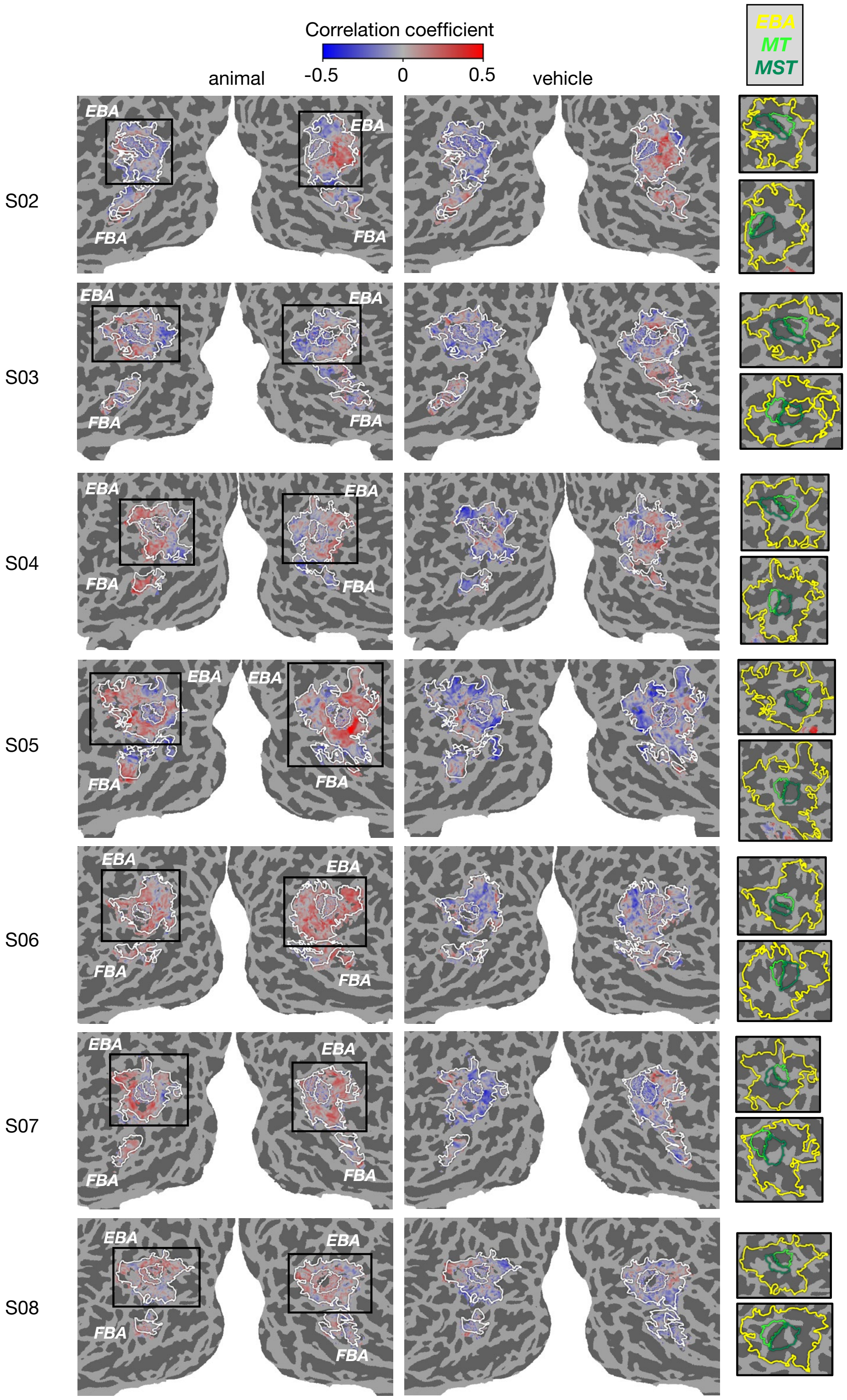

Supplementary Figure 8. Cortical surface maps of the correlation between vertex-wise responses and implied motion ratings of animal and vehicle images for the remaining seven NSD subjects. The insets on the right indicate the locations of overlapping regions: the EBA (yellow) and motion-selective regions (light and dark green; MT and MST). The maps for subject 01 are shown in Figure 5A.
